# Trends in generalised anxiety disorders and symptoms in primary care: UK population-based cohort study

**DOI:** 10.1192/bjp.2020.159

**Published:** 2021-03

**Authors:** April Slee, Irwin Nazareth, Nick Freemantle, Laura Horsfall

**Affiliations:** 1 Department of Primary Care and Population Health, University College London, UK; 2 Comprehensive Clinical Trials Unit, University College London, UK

**Keywords:** Anxiety disorders, depressive disorders, out-patient treatment, primary care, epidemiology

## Abstract

**Background:**

Generalised anxiety disorder and symptoms are associated with poor physical, emotional and social functioning and frequent primary and acute care visits. We investigated recent temporal trends in anxiety and related mental illness in UK general practice.

**Aims:**

The aims of this analysis are to examine temporal changes in recording of generalised anxiety in primary care and initial pharmacologic treatments.

**Method:**

Annual incidence rates of generalised anxiety diagnoses and symptoms were calculated from 795 UK general practices contributing to The Health Improvement Network (THIN) database between 1998 and 2018. Poisson mixed regression was used to account for age, gender and general practitioner practice. Subsequent pharmacologic treatment was examined.

**Results:**

Generalised anxiety recording rates increased in both genders aged 18–24 between 2014 and 2018. For women, the increase was from 17.06 to 23.33/1000 person years at risk (PYAR); for men, 8.59 to 11.65/1000 PYAR. Increases persisted for a composite of anxiety and depression (49.74 to 57.81/1000 PYAR for women; 25.41 to 31.45/1000 PYAR for men). Smaller increases in anxiety were seen in both genders age 25–34 and 35–44. Anxiety rates among older patients remained stable, although a composite of anxiety and depression decreased for older women. About half of drug-naïve patients were prescribed anxiety drugs within 1 year following diagnosis. The most common choice was a selective serotonin reuptake inhibitor. Benzodiazepine prescription rate has fallen steadily.

**Conclusions:**

We observed a substantial increase in general practitioner consulting for generalised anxiety and depression recently, concentrated within younger people and in particular women.

Generalised anxiety disorder is defined by worry that has exceeded its usefulness as a motivating force and become counterproductive and debilitating.^1^ The day-to-day challenges for people with generalised anxiety include reductions in physical, emotional and social functioning.^2^ In addition, generalised anxiety is a burden to the healthcare system because of frequent primary and acute care visits,^1,3^ and missed work.^4^ Most of the common mental disorders in the UK, including generalised anxiety disorder, are identified and managed in primary care settings.^5^ Generalised anxiety and depression are frequently comorbid: about 62% of patients with generalised anxiety disorder also had at least one episode of major depressive disorder during their lifetime.^6^ This finding is probably related to both a predisposition to mental illness and the substantial overlap in the diagnostic criteria for these disorders. Diminished ability to think or concentrate, restlessness and sleep disturbance appear in the DSM-V criteria for both generalised anxiety and major depression.^7^

There are few studies examining temporal trends in generalised anxiety and depression in UK general practice since 2010, and substantial variability in code lists makes comparison of rates across studies difficult.^8^ In consideration of the relationship between generalised anxiety and depression, we examined the rates of anxiety as well as a composite of generalised anxiety and depression disorders and symptoms in UK general practice over the 20-year period, from 1998 to 2018. We also explored differences by age and gender. The National Institute for Health and Care Excellence (NICE) guidelines for treatment of generalised anxiety were first published in 2011, and key recommendations included sertraline, a selective serotonin reuptake inhibitor (SSRI), as first-line therapy and short-term use of benzodiazepines for acute anxiety only, because of their potential for addiction and misuse. To examine temporal changes and alignment with these guidelines, we examined patterns of drug prescriptions among patients diagnosed with generalised anxiety disorders.

## Method

### Data source

Data from 795 general practitioner practices contributing clinical consultations data to The Health Improvement Network (THIN) were used for this analysis. THIN is a longitudinal database in which general practitioners enter medical diagnoses and symptoms as Read codes,^9^ which are cross-linked to the ICD diagnosis system and form a hierarchical coding system used to record clinical findings. Therapeutic prescriptions are also recorded. The THIN database provides a record of all clinical encounters relating to patients registered with a practice. In the UK, 95% of general practices record their clinical details on computers and do not maintain paper records. Any person registering with the practice at the point of registration offers consent for their clinical data to be recorded on these computers. Thus, the THIN database is a complete clinical record of all people registered with the practice. It includes practices spread throughout the UK. THIN contains data for approximately 6% of the UK population. The THIN database has been found to be generalisable to the UK for demographics and major condition prevalence.^10^ THIN is supplied by IQVIA Medical Research Data.

### Ethical approval

Ethical approval to use the THIN database was received on 21 May 2019 from the University College London Scientific Review Committee (reference number 19THIN029). THIN is a registered trademark of Cegedim SA in the UK and other countries. References made to the THIN database are intended to be descriptive of the data asset licensed by IQVIA. This work uses de-identified data provided by patients as a part of their routine primary care.

### Participant informed consent

Patients are informed that their data are collected for scientific research by means of posters in their general practitioner practice. They can withdraw their consent at any time by notifying the practice. All records for any patients that have withdrawn consent are removed from the THIN database.

### Study design and patient selection

Records from 1 January 1998 to 31 December 2018 were used in this cohort study because a previous analysis of the THIN database examined trends in generalised anxiety from 1998 to 2008, and we wanted to confirm that the trends over the first 10-year period were consistent with previous findings now that the number of practices participating in THIN has doubled.^11^ Patients born in or after 1910 were selected for the analysis cohort. Cohort entry occurred when patients were at least 18 years of age, had been registered with their general practice for at least 12 months (to avoid counting historical diagnoses made at a different practice)^12^ and were registered at a general practice that met prespecified quality standards.^13, 14^ Patients were censored at the last date of data collection from the general practice, date of transfer to a different practice, date of death or 31 December 2018, whichever date was earliest. Patients with fewer than 12 months between cohort entry and exit dates were excluded so that all patients had a minimum of 1 year of follow-up data that met pre-defined quality standards. Patients with a diagnosis of generalised anxiety disorder, generalised anxiety symptoms, depression, depression symptoms or mixed anxiety and depression before cohort entry were excluded.

### Diagnosis and prescription identification

Read code lists for generalised anxiety states, generalised anxiety symptoms and mixed anxiety/depression states were based on a previous analysis.^11^ A Read code list for depression and depression symptoms was based on previous work with THIN,^15^ with diagnoses for specific situational depression (e.g. postoperative depression, postnatal depression) excluded. Only medical codes and not prescription drugs were used to define cases. The complete Read code list can be found in Supplementary Appendix 1 available at https://doi.org/10.1192/bjp.2020.159.

Prescription drugs were analysed by class. Antipsychotics, tricyclic antidepressants (TCAs) and SSRIs were classified according to the British National Formulary^16^ coding assigned by the general practitioner (BNF chapter and section 4.2.1 excluding prochlorperazine, 4.3.1, and 4.3.3, respectively). This strategy of classification by BNF chapter has been used for at least one previous analysis in THIN,^17^ and has the advantage of capturing the intent of the prescription; the inclusion of prescribing for off-label indications should be reduced with this approach. Serotonin–norepinephrine reuptake inhibitors (SNRIs) were defined to include venlafaxine and duloxetine classified as section 4.3.4: Other antidepressant drugs (duloxetine has other uses, including reduction of neuropathic pain). Benzodiazepines were classified as any benzodiazepine-derivative coded to section 4.1.2: Anxiolytics (to exclude other indications such as alcohol withdrawal and neurological disorders). The drugs and classifications included in this analysis can be found Supplementary Appendix 2.

### Statistical analysis

Duration of cohort inclusion was calculated as the time from cohort entry to cohort exit. Annual incidence rates were calculated by dividing the annual number of incident cases by the total person-years at risk (PYAR) for each year. The primary interest of this analysis is generalised anxiety, defined as recording of a generalised anxiety disorder or generalised anxiety symptoms. As a sensitivity analysis, the rates for any of generalised anxiety, depression (including a diagnosis of depression or depression symptoms) or mixed anxiety and depression were calculated. Rates were stratified by age group and gender.

A Poisson mixed effects model was fitted to the counts of diagnosis of generalised anxiety or generalised anxiety symptoms, using a log-link function and with the log of the number of diagnoses during that year as an offset (weighting) variable. Age group, gender, time (as the number of years since 1997 and parameterised as a linear effect) and the two-way multiplicative interactions of these covariates (age group×gender, age group×time, gender×time) were included as fixed effects. In addition to the linear fixed effect of time, an approximate random effects low rank smoother for time within practices was included to account for time trends.^18^ The results were exponentiated to transform them back to the rate scale. The SAS macro %NLEstimate for Windows,^19^ which uses the delta method^20^ to calculate the variance of functions of parameters from the variance/covariance matrix, was used to calculate the s.e. of the rates for each age group, gender and year. Rate differences and confidence intervals comparing the most recent 4 years (2014–2018) were also calculated with %NLEstimate. Because there is overlap in the diagnostic criteria for generalised anxiety and depression, which may result in misclassification,^21^ we also considered a composite rate of generalised anxiety symptoms, generalised anxiety diagnosis, depression symptoms, depression diagnosis or mixed anxiety and depression as a sensitivity analysis.

The main goal of the prescription drug analysis was to determine prescribing trends for generalised anxiety (diagnosis or symptoms), so a cohort of patients with a generalised anxiety diagnosis and at least 1 year of follow-up after diagnosis was constructed. For each drug class (antipsychotics, TCAs, SSRIs, SNRIs, benzodiazepines), patients with any prescription in the 1 year before generalised anxiety diagnosis were excluded. Patients were classified based on year of generalised anxiety diagnosis, and the proportion of patients receiving each class of drug was calculated.

Analyses were performed with Stata version 15, SAS version 9.4 and R version 3.6.2, all for Windows.

## Results

**Fig. 1:**
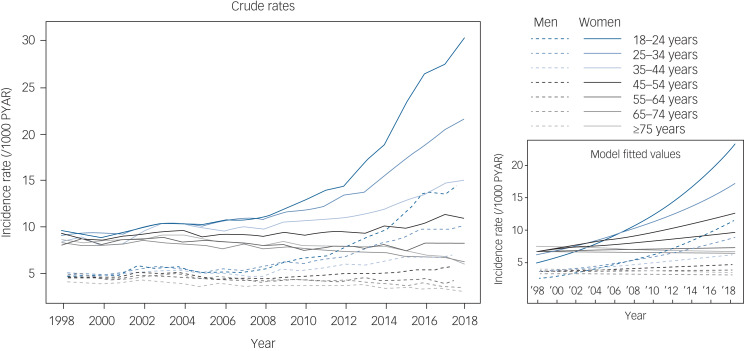
Recording of generalised anxiety diagnoses or symptoms by age group and gender. Annual incidence rates (left) for generalised anxiety diagnoses or symptoms were calculated by dividing the annual number of incident cases by the total person-years at risk (PYAR) for each year. Model fitted values (right) were calculated from a Poisson mixed effects model with a time smoother.

Among the 6 630 040 patients who met the inclusion criteria for this analysis, there was a total of 52 695 827 PYAR. There were 400 667 generalised anxiety diagnoses. Of these, 151 339 (37.8%) recorded generalised anxiety disorder only, 203 703 (50.8%) recorded generalised anxiety symptoms only, and 45 625 (11.4%) recorded both generalised anxiety diagnosis and generalised anxiety symptoms. For generalised anxiety diagnoses and symptoms, the four most common Read codes were ‘Anxiety states’ (43.4%), ‘Anxiousness – symptom’ (27.9%), ‘Anxiousness’ (17.0%) and ‘Anxiety state NOS’ (4.1%). These four codes accounted for 92.5% of patients with generalised anxiety diagnoses or symptoms identified. The crude generalised anxiety rates over time and model fitted values (prediction) are shown in Fig. 1. The rate of generalised anxiety diagnoses was higher for women compared with men across all age groups. Generalised anxiety rates from 2014 to 2018 show sharp increases for patients aged 18–24 years and 25–34 years; this pattern holds for both men and women, but is especially pronounced in young women. In contrast, the generalised anxiety rates among patients aged ≥55 years do not mirror the increases seen in younger patients. These older patients show recent stability or slight decreases in generalised anxiety rates.

**Fig. 2:**
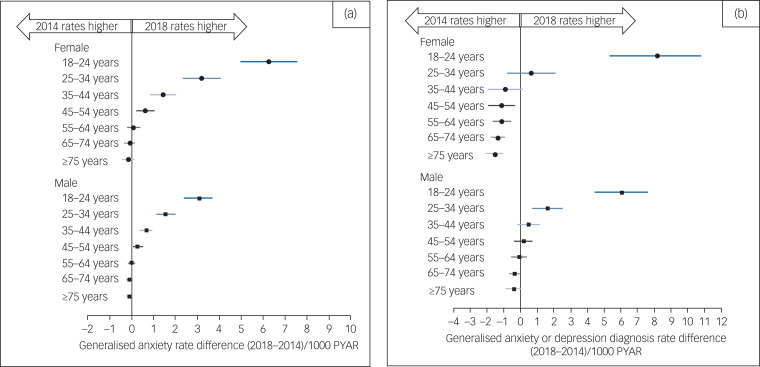
Change in incidence of generalised-anxiety-related diagnosis or symptoms from 2014 to 2018, by age group and gender. (a) Recording generalised anxiety diagnosis or symptoms. (b) Recording generalised anxiety, depression or mixed disorders or symptoms. Difference between 2014 and 2018 incidence is calculated from a Poisson mixed effects model. 95% confidence intervals were calculated with the delta method. Values to the right of zero indicate an increase in generalised anxiety recording, and values to the left of zero indicate a decrease in recording in primary care. PYAR, person-years at risk.

Details of the recent changes in generalised anxiety rates can be seen in Fig. 2(a), which shows model-based rate differences between 2014 and 2018 by age and gender. Among women, all age groups aged ≤54 years have shown significant increases in generalised anxiety rates since 2014. The increase among women aged 18–24 years is about twice the increase for the next youngest cohort of women and for the youngest group of men. Generalised anxiety rates for patients aged ≥55 years are largely unchanged. Crude rates and model-based estimates with s.e. can be found in Supplementary Appendix 3. Displays of time trends in generalised anxiety based on symptoms and generalised anxiety based on diagnoses can be found in Supplementary Appendix 5.

**Fig. 3:**
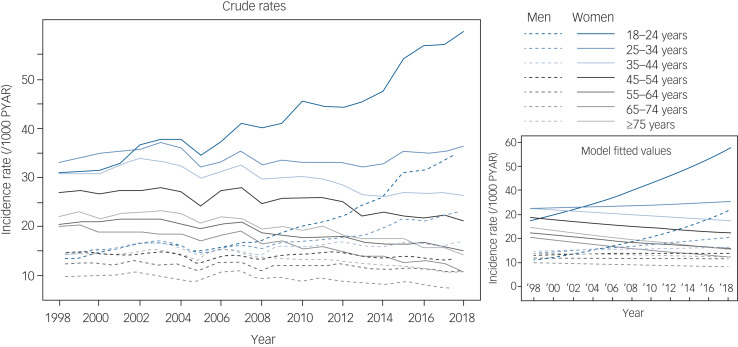
Recording of generalised anxiety, depression or mixed disorders or symptoms, by age group and gender. Annual incidence rates (left) for patients with a recorded generalised anxiety disorder, generalised anxiety symptoms, depression, depression symptoms or mixed anxiety and depression were calculated by dividing the annual number of incident cases by the total person-years at risk (PYAR) for each year. Model fitted values (right) were calculated from a Poisson mixed effects model with a time smoother.

This methodology was repeated for any diagnosis of depression, depression symptoms, generalised anxiety, generalised anxiety symptoms and mixed anxiety and depression as a sensitivity analysis. There were 989 750 patients with at least one of these diagnoses recorded. Among them, 224 593 (22.7%) recorded generalised anxiety diagnosis or symptoms only, 513 050 (51.8%) recorded depression or depression symptoms only, 37 300 (3.8%) recorded mixed anxiety and depression only, and 214 807 (21.7%) recorded more than one of these categories. Crude rates by age and gender are displayed in Fig. 3. Considering all generalised anxiety and depression diagnoses, the rates are relatively stable, with the exception of the youngest group of women (18–24 years) and the two youngest groups of men (18–24 years and 25–34 years). For all age groups, the rates of any generalised anxiety or depression diagnosis are higher among women than men.

Similar to the analysis for generalised anxiety alone, the analysis of recent changes in generalised anxiety, depression or mixed recordings can be seen in Fig. 2(b), which shows model-based rate differences between 2014 and 2018 by age and gender. The youngest age groups for both men and women show dramatic recent increases in any depression or generalised anxiety diagnosis, and there is a more modest increase among men aged 25–34 years. The combined rate has fallen for women aged ≥45 years, and is relatively unchanged for men aged ≥35 years. Crude rates for any generalised anxiety or depression diagnosis and model-based estimates with s.e. can be found in Supplementary Appendix 4.

**Fig. 4:**
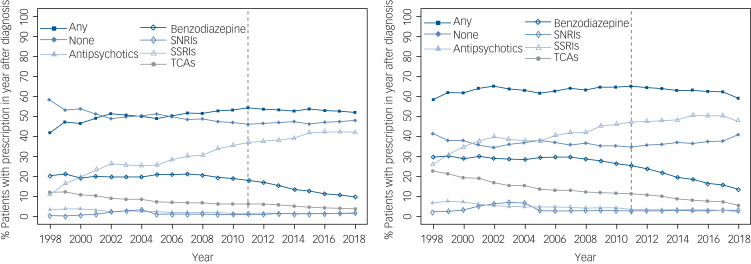
Proportion of patients with generalised anxiety recording by first treatment strategy after diagnosis. Percentages were calculated by dividing the total number of patients with at least one prescription for each medication class in the year following first generalised anxiety recording by the number of initial generalised anxiety recordings for that year. The left panel shows percentages for patients with no generalised anxiety prescription in the year before general practitioner recording, and the right panel shows the percentages for all incident generalised anxiety cases regardless of prescriptions in the year before first recording. SNRIs, Serotonin–norepinephrine reuptake inhibitors; SSRIs, selective serotonin reuptake inhibitors; TCAs, tricyclic antidepressants.

Initial treatment for generalised anxiety disorders and symptoms is shown in Fig. 4. These figures show the proportion of patients with generalised anxiety disorders and generalised anxiety symptoms recorded through 1 year after diagnosis, and at least 1 year of follow-up after the first generalised anxiety diagnosis is required for inclusion in this analysis. The left panel is the subset of patients who were not taking any psychotropic medication for generalised anxiety (antipsychotics, benzodiazepines, SNRIs, SSRIs and TCAs) in the year before generalised anxiety diagnosis, and the right panel is all patients with 1 year of follow-up after diagnosis, regardless of previous prescriptions. As patients could be treated with more than one medication in the year following the first recording of generalised anxiety, the proportion of patients taking any medication and no medication is also shown.

Among patients not taking psychotropic medication before generalised anxiety diagnosis, about half receive drug treatment in the first year. There has been a slight increase in the proportion receiving medication since about 2006. The rates of benzodiazepine use began decreasing around 2008, and this decrease has continued through 2018. The proportion using SSRIs has increased steadily over the study period.

## Discussion

To the best of our knowledge, this analysis is the largest and most recent assessment of time trends for general anxiety in general practice. This is likely because of the general lack of large, standardised primary care data-sets. The most important finding of this analysis is the sharp increase in the recording of generalised anxiety disorders and symptoms in the past few years, and the disproportionate impact of this increase on young adults. Recording rates for the combination of generalised anxiety, depression or mixed have been relatively stable, except for sharp increases for men and women aged 18–24 in the past 10 years, and a moderate increase for men aged 25–34 years in the past 4 years. Although the rates of incident recordings for generalised anxiety are about twice as high for men compared with women across age cohorts, the rates of the recent increases in the youngest cohorts are parallel across genders, suggesting that the increases in rates over time are similar. For women aged 35–44 years and 45–54 years, the recent increase in generalised anxiety corresponds with a decrease in the composite of generalised anxiety, depression or mixed. Taken together, these findings could illustrate a difference in general practitioner recording patterns. However, for the cohorts of women aged ≥55 years, there has been no increase in generalised anxiety and, simultaneously, a decrease in the composite, suggesting temporal changes in depression for older women. For men aged 18–24 years and 25–34 years, both generalised anxiety and the composite were increased, which is inconsistent with a change in reporting patterns. The trends since about 2008 are largely similar for anxiety diagnoses and anxiety symptoms, although from 1998 to 2008 there were declines in diagnoses and slight increases in anxiety symptom recording across all strata. This could be explained by change in coding practice by the general practitioners over this period, which seemed to stabilise thereafter.

Consistent with previous reports,^22–24^ this analysis demonstrates generalised anxiety is much more commonly reported in women than men. To the best of our knowledge, the recent substantial increase in generalised anxiety in younger cohorts has not been previously reported in a UK general practice setting, which is of considerable public health importance. This increase may reflect an underlying increase in generalised anxiety in the general population or an increasing awareness of, and investigation for, generalised anxiety on the part of general practitioner, although the concentration of the increase in the younger adults may mitigate against a change in general practitioner behaviour being a plausible explanation for these observations. Although the increase is most striking in young women, there has also been a significant recent increase in generalised anxiety recording for young men. The median healthcare cost for persons with generalised anxiety disorder are estimated to be 64% higher than for those without generalised anxiety disorder,^25^ so, along with considerable health decrement, this recent increase in recording is likely to cause a material depletion of budgeted resources. Although the increases were similar across genders, the budget and public health impact of the increase for women will be greater than for men, because of the initial rate differences. Primary care clinicians may usefully be aware of these increases and remain vigilant for generalised anxiety disorders and symptoms in all young adults.

Changes in suicide rate in the UK are consistent with the changes in generalised anxiety and depression. Correlates of suicide that may also interact with mental illness include psychosocial crisis, hopelessness, impulsivity, treatment and many others. Although young people represent a small number of total suicide deaths, the suicide rates among 10- to 24-year-olds have increased recently for both genders, according to the 2018 report from the Office for National Statistics. There was a significant increase of 31% from 2017 to 2018 among males aged 20–24 years. Among females aged 10–24 years, the suicide rate in 2018 was 3.3 deaths/100 000 females, which is the highest rate since recording began in 1981. Comparing the changes from 2014 to 2018, there have been increases in the two youngest age cohorts, with the increase in the 10–24 years cohort about twice the increase in the 25–44 years cohort.^26^ Although suicide is more common in men than women across all age groups, self-harm is more common in women. A recent study of adolescents in the UK found a 68% increase in self-harm among girls aged 13–16 years, from 45.9 per 10 000 in 2011 to 77.0 per 10 000 in 2014.^27^ A study based on data from the National Self-Harm Registry in Ireland found increases between 2007 and 2016 in self-harm presenting at hospital emergency rooms of 14% and 29% for males and females aged 10–24 years, respectively.^28^ Whether and how increases in generalised anxiety and depression relate to increases in self-harm and suicide is unclear, but it is clear that increases in both psychiatric and behavioural changes are affecting young people, and young women in particular.

It is notable that rates of generalised anxiety disorders and symptoms began their current upward trajectory around the time that the effects of the 2008 economic downturn and during the policy of austerity. The relationship between employment,^29,30^ finances^31,32^ and anxiety is well known. Of note, generalised anxiety is also taking a toll on employed people; the Office for National Statistics reported a 24% increase between 2009 and 2014 in the number of sick days lost to ‘stress, depression and anxiety’ in England and Wales.^33^

The difference in generalised anxiety rates by age probably reflect several recent changes. Social media use, which early research suggests is strongly associated with anxiety, is more common among young people and may be partly responsible. A recent study in 563 emerging adults (aged 18–22 years) found that daily social media use was associated with dispositional anxiety scores indicating a probable anxiety disorder.^34^ Profitable social media platforms use a business model that monetises the attention of users by selling it to advertisers, but in actuality, social media users are the product and not the customers. Maximising profit is analogous to maximising the amount of time that users spend engaged with the platform, which can interfere with personal and professional responsibilities. For example, in the USA, a study of 1730 adults aged 19–32 years identified five categories of social media use. The two highest levels (labelled ‘wired’ and ‘connected’) had odds ratios of 1.9 and 3.1 for depression and 4.5 and 2.2 for anxiety symptoms compared with the lowest level (‘unplugged’).^35^ However, it is unclear to what extent these relationships may be causal or, alternatively, an alternate manifestation of the underlying anxiety. In addition, cuts to social services may disproportionately affect young people, as housing, university or apprenticeship opportunities, and employment are less certain under the recent austerity paradigm. These uncertainties may disproportionately increase generalised anxiety in young people who are seeking to establish careers, and have minimal effect on people with well-established careers and retired people.

Previous research has described the link between anxiety and drug use.^36^ Increasing anxiety rates are temporally associated with changes in drug and alcohol use in young adults in the UK. However, the increases in anxiety in this analysis are occurring against a backdrop of recent reductions in alcohol use among young adults. A longitudinal assessment of 10 000 people aged 16–24 years showed an increase in the proportion of non-drinkers and a reduction in the rate of binge drinking from 2005 to 2015.^37^The Office for National Statistics Opinions and Lifestyle Survey also found recent reductions in alcohol consumption, and especially among 16- to 24-year-olds.^38^ The trends are more ominous for illicit drug use. An international 7-year analysis of wastewater demonstrated higher levels of cocaine (based on benzoylecgonine levels) and 3,4-methylenedioxy-methamphetamine (MDMA, ecstasy) consumption in the UK in 2014–2017 compared with 2011–2013.^39^ The Office for National Statistics reports that illicit drug use in the UK for adults aged 16–59 years has remained relatively stable over the past decade, although there has been a slight increase recently.^40^ Young adults aged 16–24 years appear to be responsible for this increase, and higher incidences of powder cocaine and ecstasy use were reported in this age group. Drug use is lower for women compared with men, but the difference in rates of use is largely unchanged since 2008.

Although we continue to study the underlying reasons for increasing rates of generalised anxiety, there are several effective therapeutic^41^ and psychological^42^ treatments for generalised anxiety disorder, although how these treatment options perform on a group of patients identified by symptoms rather than generalised anxiety disorder diagnoses has not been studied. One of the key messages in the 2011 NICE generalised anxiety guidelines was the recommendation not to prescribe benzodiazepine as a treatment for generalised anxiety disorder, and to limit its duration of use to 1–2 weeks. We did not examine duration in this study, but we did identify a reduction in benzodiazepine prescribing among psychotropic-naïve (at least in the past year) patients. Sertraline, an SSRI, is the recommended first-line therapy, and the proportion of patients diagnosed with generalised anxiety who received an SSRI prescription increased, to become the most common therapeutic category over the course of the study. The increase in SSRI use and reduction in benzodiazepine use began before the publication of the NICE guidelines,^5^ but since the release of the guidelines, both trends have continued. When patients taking medication before recorded generalised anxiety were considered, these patterns were largely similar. The use of TCAs was more common at the beginning of this study, but there has been a steady reduction over time. These findings suggest that general practitioners are aware of the NICE guidelines, and prescribing patterns are more adherent to these recommendations now than at any prior time point.

This analysis has several limitations. First and foremost, it is based on identification of symptoms and diagnoses in patients seeking help from their general practitioners. We selected data by using the practice specific quality indicators of acceptable computer usage^14^ and mortality reporting,^13^ which should improve the accuracy of the data in our analysis, but may slightly reduce generalisability. Although there is evidence for the accuracy of recorded diagnoses among those who seek help,^43^ these results will underestimate the incidence rates of generalised anxiety and depression in the UK because many patients experiencing generalised anxiety and depression do not seek help, or do not seek help from their primary care general practitioner. However, this analysis does represent a broad group of patients who do present to their general practitioners seeking help. As described above, there is some evidence of hesitation on the part of general practitioners to record generalised anxiety disorders even when suspected clinically, and to instead report generalised anxiety symptoms. The reluctance to record mental illness may extend further, to a group presenting with generalised anxiety symptoms where even these are not recorded. In addition, we cannot determine whether changes in reporting of generalised anxiety and depression are driven by changes in the relative rates of underlying disease, in health-seeking behaviour or simply a change in the way general practitioners classify patients with an overlapping set of symptoms. However, especially in young people, the evidence of a recent increase in rates is impervious to these classification problems.

An additional limitation is that our analysis was restricted to generalised anxiety and did not examine other anxiety disorders such as panic disorder, obsessive–compulsive disorder and social phobias. Although generalised anxiety is frequently comorbid with other anxiety disorders, the context and management of generalised anxiety differ from other anxiety disorders, and this work is insufficient to address these nuances adequately. Our analysis also did not account for concurrent substance misuse, which is also comorbid with anxiety disorders but tends to be poorly coded in general practice.^44^

## Data Availability

The data that support the findings of this study are available from IQVIA. Restrictions apply to the availability of these data, which were used under license for this study. Data are available from the authors with the permission of IQVIA.

## Supplementary material

For supplementary material accompanying this paper visit https://doi.org/10.1192/bjp.2020.159. click here to view supplementary material
