# Supplementary material for: Trends in generalised anxiety disorders and symptoms in primary care: UK population-based cohort study
Source: Br J Psychiatry. 2021 Mar;218(3):158–64. doi: 10.1192/bjp.2020.159 (PMC8529638; doi:10.1192/bjp.2020.159)
Supplement: Supplementary file 1 [file S0007125020001592sup.zip › S0007125020001592sup001.docx]

Contents

[Supplementary Appendix 1: Read Code Lists 2](#_Toc42087223)

[Supplementary Appendix 2: Prescription Classifications 4](#_Toc42087224)

[Supplementary Appendix 3a: Anxiety recording in primary care (diagnosis or symptoms): Female 5](#_Toc42087225)

[Supplementary Appendix 3b: Anxiety recording in primary care (diagnosis or symptoms, cont'd): Male 6](#_Toc42087226)

[Supplementary Appendix 4a: Anxiety, Depression or Mixed recording in primary care (diagnosis or symptoms): Female 7](#_Toc42087227)

[Supplementary Appendix 4b: Anxiety, Depression or Mixed recording in primary care (diagnosis or symptoms, cont'd): Male 8](#_Toc42087228)

[Supplementary Appendix 5: Comparison of trends in anxiety based on symptom codes and diagnosis codes 9](#_Toc42087229)

# Supplementary Appendix 1: Read Code Lists

**Supplementary Appendix 1: Read Code Lists (cont'd)**

# Supplementary Appendix 2: Prescription Classifications

# Supplementary Appendix 3a: Anxiety recording in primary care (diagnosis or symptoms): Female

# Supplementary Appendix 3b: Anxiety recording in primary care (diagnosis or symptoms, cont'd): Male

# Supplementary Appendix 4a: Anxiety, Depression or Mixed recording in primary care (diagnosis or symptoms): Female

# Supplementary Appendix 4b: Anxiety, Depression or Mixed recording in primary care (diagnosis or symptoms, cont'd): Male

# Supplementary Appendix 5: Comparison of trends in anxiety based on symptom codes and diagnosis codes


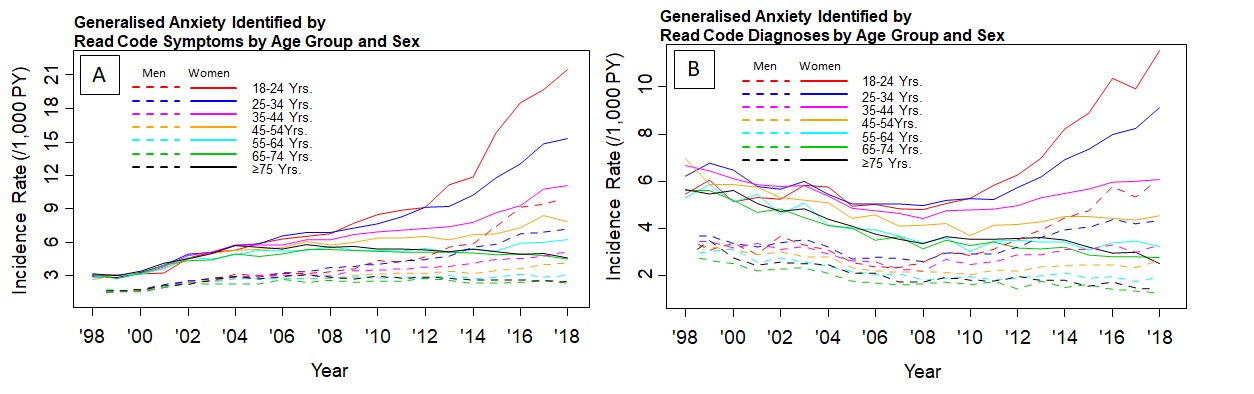


Panel A – Generalised anxiety identified by symptoms (e.g. “Anxiousness”, “Tension – nervous”). Panel B – Generalised anxiety identified by diagnoses (e.g. “Anxiety State”, “Chronic Anxiety”).
